# Legitimizing incapacity: discursive choices in Norwegian sickness certificates

**DOI:** 10.1186/s12913-025-12902-7

**Published:** 2025-05-20

**Authors:** Egidio Niclas D’Angelo, Ralf Kirchhoff, Kristin Halvorsen

**Affiliations:** 1 Department of Health Sciences, Ålesund, Faculty of Medicine and Health Sciences, Norwegian University of Science and Technology (NTNU), Ankeret, A316, Ålesund, Norway; 2 Department of Language and Literature, Faculty of Humanities, Bygg 5, 5519A, Dragvoll, Trondheim, Norway

**Keywords:** Sickness certificates, Discourse analysis, Patient work ability, General practitioners, Norway, Mental health, Welfare assessments

## Abstract

**Background:**

In Norway’s welfare system, General Practitioners (GPs) issue sickness certificates (SCs) to document patient’s inability to work. These documents serve a dual role as medical evidence and as a basis for social welfare decisions. The language used in SCs can shape how non-medical stakeholders perceive a patient’s work capacity. This study examines how SC language constructs narratives of work ability, focusing on how it portrays patients’ limitations and prospects for recovery.

**Methods:**

We conducted a qualitative discourse analysis of 155 SCs written by Norwegian GPs for patients under 35 years old with common mental health conditions. We focused on certificates issued around week 39 of the patient’s sick leave. Using discourse analysis techniques, we examined linguistic features that convey the patient’s work capacity and functional limitations.

**Results:**

SCs predominantly emphasized incapacity and the necessity of work absence through discursive choices such as definitive language, amplified descriptions, and rhetorical strategies reinforcing limitations. Recovery potential was presented with tentative language, reflecting uncertainty in prognosis, while the temporal dimension of treatment was frequently framed as a barrier to returning to work. Additionally, the use of specialized terminology, generalized label, and elliptical constructions placed a significant interpretative burden on non-medical readers. Furthermore, SCs largely lacked explicit recommendations for workplace accommodations or interdisciplinary collaboration, limiting their utility in facilitating structured return-to-work strategies.

**Conclusions:**

Time constraints, administrative pressures, and the dual roles of GPs as clinicians and bureaucrats shape the entire production of SCs. In turn, these discursive choices often reinforce narratives of incapacity. Enhancing SC relevance through structural modifications and interdisciplinary collaboration, including employer involvement in evaluating workplace accommodations, could improve welfare assessments and support tailored reintegration strategies. Positioning SCs as collaborative tools – rather than standalone assessments – may better align clinical evaluations with workplace realities and foster shared accountability for recovery and return-to-work efforts. SCs seem to place a disproportionate burden on GPs to translate medical conditions into work-related recommendations, often without the support or expertise required for such interdisciplinary evaluations.

**Supplementary Information:**

The online version contains supplementary material available at 10.1186/s12913-025-12902-7.

## Background

Sickness certificates (SCs) are key documents within public healthcare and welfare systems. They serve two main purposes: they confirm patients’ inability to work due to health conditions and provide information about their functional abilities. In Norway’s healthcare model, SCs communicate medical assessments and are crucial in securing patient access to financial and social support [1]. Typically issued by general practitioners (GPs), SCs support both patient care and administrative requirements of Norway’s healthcare and welfare systems, making them a crucial link between healthcare systems and the Norwegian Labor and Welfare Administration (NAV) in Norway [2].

NAV relies on SCs to evaluate patients’ eligibility for welfare benefits, determine necessary support levels, and identify accommodations for returning to work [1–5]. By serving as the primary source of information about a patient’s disabilities, they are a key tool for conveying information between physicians in the healthcare systems and social workers in NAV, ensuring decisions by NAV on sickness benefits are justified. Incomplete or ambiguous information on SCs, particularly regarding functional capacity, can delay decisions and hinder timely return-to-work measures [4]. Consequently, how SCs are phrased and structured can influence decision outcomes, impacting decisions about the timing and extent of support [6, 7].

In Norway’s healthcare system, GPs serve as gatekeepers, managing patient access to specialized services and coordinating healthcare, with an inherent emphasis on resource-conscious policy making to ensure universal access ([8], pp. 159–168). Legislative changes, evolving documentation standards and growing service expectations have expanded the administrative workload of Norwegian GPs. Balancing clinical care with responsibilities like issuing medical documentation places GPs under substantial pressure to meet both patient and institutional demands [9, 10].

SCs often place greater emphasis on incapacity rather than recovery potential, as indicated by trends observed in previous research [11], and this can bias welfare decisions toward extended support. GPs may face systemic pressures, including patient expectations and waiting times, which may lead to longer sick leave periods than strictly medically justified [12]. These challenges are particularly acute in mental health cases, where subjective symptoms and the interplay of psychological, social, and occupational factors complicate work capacity assessments. In such contexts, conflict and role strain can arise for GPs when external pressures outweigh purely clinical judgments, reinforcing a narrative of incapacity in SCs [13].

The current study addresses a gap in the empirical examination of SCs by analyzing authentic SC texts submitted to NAV and produced by Norwegian GPs. Using J.P. Gee’s discourse analysis framework [14], we conduct an in-depth exploration of how language constructs narratives of incapacity and recovery, moving beyond institutional interpretations or outcomes to illuminate GP textual practices involved in portraying (un)health and work ability.

### The Norwegian healthcare landscape

In Norway, employees are entitled to up to 52 weeks of paid sick leave. GPs are typically responsible for issuing SCs when a patient’s health condition is deemed incompatible with work duties to some degree. Depending on the duration of the leave, specific checkpoints (at 7, 17 and 39 weeks of sickness duration) require more detailed evaluations from the GP. The SC consists of standardized forms where the GP documents the medical basis for incapacity and, ideally, recommendations for workplace adjustments. These certificates are then submitted to NAV, whose social workers assess eligibility for benefits and plan potential return-to-work measures. While NAV employs *rådgivende overleger* (senior consulting physicians) to advise on complex cases, these physicians review only a fraction of all certificates. Consequently, social workers – most of whom lack formal medical training – still rely heavily on the clarity and accessibility of the SC itself to guide their decisions.

This creates an interdisciplinary challenge, where SCs must translate complex medical assessments into language accessible for welfare evaluations. Similar challenges in healthcare-welfare collaboration have been observed in Sweden, where communication gaps between healthcare and welfare led to delays and misunderstanding [15]. These findings highlight the need for knowledge about communicative and textual practices in the intersection between health and welfare.

### Bio-medical emphasis in SCs

Sturesson et al. [3] note that the multifaceted purpose of medical certificates often leads to difficulties in crafting language that satisfies both legal and practical needs, particularly for employers and patients. However, content analysis of SCs from Sweden reveal that they often prioritize diagnoses over functional descriptions, relying on concise technical language to meet institutional requirements [16]. Similarly, another paper highlights that the use of definitive phrasing and technical language can create barriers in communication, reinforcing hierarchical structures that may limit patient engagement [4]. A purely bio-medical focus can complicate the process of certifying work incapacity. From the patient side, the wording of a sickness certificate can influence autonomy, legitimacy and engagement with rehabilitation; clearer, capacity-focused language has been shown to enhance patients’ sense of agency in the return-to-work process [17].

In a survey conducted among occupational physicians in the UK, Vivian [18] found that assessments of fitness to work often emphasized incapacity over work potential. This underscores the difficulty of incorporating psychological and social factors – central to the biopsychosocial model – into incapacity evaluations, which have historically leaned toward purely bio-medical assessments. While this study specifically examines occupational physicians, the findings may parallel challenges faced by general practitioners, who similarly navigate the tension between clinical judgments and broader psychosocial considerations in certifying work capacity. Our previous work [11] has confirmed a predominant use of bio-medical language and focus on patient limitations over abilities in SCs, thus reinforcing the concerns noted in previous studies.

As Timpka et al. [19] observe, physicians must move beyond the traditional clinical role to integrate social and occupational factors into their medical assessments. In practice, this means that a diagnosis alone may be insufficient: documenting how a patient’s condition intersects with workplace demands or social circumstances is crucial. Without this broader perspective, the language used in SCs can become ambiguous or incomplete, making it harder for social workers to determine appropriate support measures.

### Bureaucratic discretion and systemic pressures

Lipsky’s theory of street-level bureaucrats [20] provides a valuable framework for understanding how frontline workers operate under conditions of limited resources, high demand, and competing priorities. Public servants – such as GPs in Norway – are delegated considerable autonomy and discretionary power, yet also face administrative mandates and accountability pressures. Within this model, Lipsky emphasizes how street-level bureaucrats use coping mechanisms to reconcile conflicting requirements, often simplifying or prioritizing certain tasks at the expense of others.

For Norwegian GPs, the sickness certification process exemplifies these pressures. Acting as both clinicians and gatekeepers, GPs must accurately assess medical conditions while adhering to formalized welfare regulations [21]. This dual role can lead to variations in documentations – some GPs may emphasize incapacity out of caution or time constraints, while others might elaborate on work potential [22]. Systemic mandates, such as NAV’s expectation of clear, justifiable claims, can overshadow more nuanced descriptions of patient functionality, thereby shaping the linguistic choices that appear in SCs. Over time, as GPs refine their documentation practices, the original complexity of an individual’s condition may be changed by administrative norms or standard phrasing.

Building on Lipsky’s insights, Zacka [23] highlights ethical dilemmas that arise when bureaucratic demands conflict with patients’ individual needs. In the context of SCs, GPs not only document incapacity but also carry a moral imperative to support a patient’s broader recovery and reintegration. Navigating these competing concerns – standardized administrative tasks versus personalized care – can foster tensions that shape how SC narratives are constructed [24]. As Shutzberg [25] describes, sick-listing consultations are shaped by both medical and bureaucratic imperatives, with GPs exercising discretionary authority in ways that often reinforce a paternalistic dynamic.

Consequently, systemic pressures influence both the form and the content of these certificates, revealing how ethical considerations intersect with the demands of street-level bureaucracy.

### Study aim

This study examines the linguistic and rhetorical choices in SCs written by Norwegian GPs, focusing on how language constructs narratives of patient work capacity and functional limitations. By identifying recurring patterns in the constructions of patients’ limitations and recovery potential, the study seeks to analyze the discursive strategies used to justify and legitimize sickness absence. These study aims to contribute to a better understanding of how language shapes the representation of patient functionality in SCs.

### Research questions

What discursive strategies are evident in sickness certificates issued by general practitioners, and how do these strategies construct narratives of work capacity and recovery potential?

## Methods

### Discourse analysis

Discourse analysis offers a valuable framework for examining healthcare documentation, particularly for understanding how language in SCs reflects and shapes healthcare practices and perceptions. As Woodward-Kron [26] illustrates, discourse analysis uncovers communication patterns within healthcare, identifying effective practices and potential barriers arising from linguistic choices. Medical discourse can encode sociocultural ideas, shaping perceptions of health and illness [27], while also mirroring cultural values within healthcare settings [28]. By analyzing linguistic elements, discourse analysis further exposes the implicit assumptions and social structures embedded in healthcare communication, potentially shedding light on how SC language may influence recipient perceptions of patient capacity [29].

J.P Gee’s discourse analysis tools [14] are particularly well-suited for analyzing SCs, as they allow for the examination of both explicit and implicit assumptions. Pedersen et al. [30] demonstrated how linguistic patterns in other medical documentation influence collaborative understanding and knowledge transfer. Building on this approach, our study applies Gee’s framework to uncover how linguistic choices in SCs construct narratives around patient functionality and work capacity. This application of Gee’s tools is valuable for examining layered meanings or intentions within SCs, providing further insights in the interdisciplinary functions of these medical texts.

### Study design and data source

This study examines the linguistic characteristics and rhetorical structures of 155 digital Norwegian SCs from patients with common mental health diagnoses, focusing specifically on SCs written in week 39 of sickness duration (template provided in Supplementary file 1). We analyzed the descriptions found under Sects. 6.x and 7.x of the SCs, where GPs respond in prose to prompts in the form/template. These sections serve critical functions in documenting functional assessments, medical history, and workplace accommodations critical for welfare assessments.

In Norway, employees can be on sick leave for 52 weeks with full pay. SCs require detailed assessment at three key points: weeks 7, 17 and 39 of sickness duration. We selected week 39 as the longer sickness period provides GPs with more time to evaluate complex mental health conditions, allowing for more comprehensive depiction of the patient’s work capacity and functional limitations. To capture a representative dataset of mental health illnesses, we selected SCs that were written for any of the six most common mental health diagnoses associated with prolonged sickness absence in Norway (Table 1).

**Table 1:** ICPC-2 (International Classification of Primary Care) classifications included in this study

P01	Feeling anxious/nervous/tense
P02	Acute stress reaction
P03	Feeling depressed
P29	Psychological symptom/other complaint
P74	Anxiety disorder/anxiety state
P76	Depressive disorder

NAV provided the SCs for the study, sourcing them from across all Norwegian counties according to the study inclusion and exclusion criteria. The SCs were anonymized by NAV before they granted researcher access. This anonymization process involved the removal of identifying information, including personal and institutional names, locations, and physician details. While gender-specific identifiers were removed, gendered pronouns in written prose were retained.

All SCs included in this study were issued between January 2018 and January 2020, with cases having already undergone assessment by NAV at the time of data collection.

#### Inclusion and exclusion criteria

We included SCs that met the following criteria:

- Patients diagnosed with mental health conditions listed in Table 1.
- Patient age under 35 at the time of sick leave.
- Sickness absence documented between 01.01.2018 and 01.01.2020
- Only one SC per patient to ensure uniformity in the dataset

We excluded SCs with the following criteria:

- SCs listing any additional diagnoses beyond those specified in Table 1. This enabled us to maintain focus on the selected mental health conditions. However, mentions of other diagnoses within free-text sections of the SCs were not grounds for exclusion, as these were not the primary reason for certification.
- Graded sick leave. We focused exclusively on 100% sick leave cases.

We focused on patients < 35 years with common mental illness because this group has an elevated risk of prolonged absence. Limiting the dataset to one certificate per patient ensured comparability and prevented any single individual’s documentation from disproportionately influencing the discourse analysis.

Each SC included all free-text sections, where GPs can provide detailed descriptions of the patient’s condition, and structured sections with checkboxes and other predefined choice options. However, we observed that some parts of the form were seldom completed, for example 7.1 *“Accommodations/considerations that should be made at the workplace. Describe (can be read by the employer”),* where only 13 out of 155 had an entry, and only 1 SC provided a substantive answer to the actual prompt. In addition, the GPs have inconsistently placed their descriptions of the patient under the various free-text headings, sometimes choosing only one place and then simply referring back to this one text under other headings. Due to these minimal and inconsistent responses to some prompts, we treated the SC free-text descriptions as one unit for analysis rather than focusing on specific sections.

### Analytic procedure

We began by conducting an initial open reading of all 155 free text sections in their original Norwegian to familiarize ourselves with the data and note any recurring linguistic patterns. Next, we applied each of the seven analytic tools (see Table 2) from Gee’s framework [14] as guiding prompts. Each tool highlights a particular aspect of discourse. We then systematically coded SCs according to these prompts, creating an initial set of thematic notes.

**Table 2:** Gee’s tools used for this study

Tools	Definition/question
1. The Subject Tool	Was used to examine how key elements, such as a patient’s diagnosis, symptoms, or work limitations, are selected and presented within the SCs. Which aspects are foregrounded or backgrounded?. It allows us to not only see what is said but also how emphasis is placed on certain issues or topics
2. The Vocabulary Tool	Was included to examine the balance between medical terminology and non-medical language, revealing how word choices might shape accessibility and readability of the information
3. The Significance Building Tool	Was used to examine how language is used to emphasize or downplay specific aspects, thereby constructing a particular narrative of work ability. It helps identify how GPs use language to boost or hedge certain aspects
4. The Figured Worlds Tool	Was applied to identify and analyze metaphors and other figurative language that constructs specific social realities around illness and recovery. By understanding the “worlds” the SCs create, we gain insights into larger discourses of mental health and disability
5. The Fill-In Tool	Was chosen to identify implicit meaning that requires non-medical readers to infer what is left unstated. While it is impossible to predict every “unknown unknown”, we flagged specialized jargon, elliptical phrasing, or implied causal links that presuppose medical knowledge not provided in the SC
6. The Doing And Not Just Saying Tool	Was employed to analyze how language not only describes a patient’s condition but also actively justifies or enacts an administrative outcome. Rather than merely emphasizing severity, as explored with Tool 3, this tool aims to identify discourse that advocates a specific course of action, effectively *doing* something beyond simply *saying* it
7. The Collaboration Tool	Was used to examine how SCs address or encourage cooperation between healthcare providers, NAV, and other stakeholders. It aims to identify gaps or strengths in the SCs’ texts that could either foster or impede actionable collaboration in welfare assessments and planning

After coding all SCs in this manner, we brought the analyses together in a cross-cutting review to identify overarching themes and points of convergence or divergence across the corpus. Finally, we translated representative excerpts into English to illustrate key findings in this manuscript while retaining their original meaning as closely as possible. This approach allowed us to draw on Gee’s tools not just to classify the data but also to interpret how specific language choices reinforce or challenge different discourses.

When presenting examples from the data, omissions in the original text are indicated by (…) at the beginning or end of sentences, signaling the presence of additional content that we chose not to display.

### Researcher reflexivity

It is important to acknowledge the primary researcher’s dual role as both a GP and a senior consulting physician at NAV. This background provides in-depth insight into the practical and systemic pressures surrounding sickness certification, potentially enriching the qualitative analysis. At the same time, it poses a risk of bias– familiarity with administrative protocols and the physician’s perspective may inadvertently shape interpretations of the SC texts. To address this, the analysis incorporated regular collaboration with the co-researchers and cross-checking interpretations against direct textual evidence. While complete neutrality is impossible, these steps enhance the trustworthiness of the findings by making researcher’s position explicit and systematically managing any resulting bias.

## Results

The free-text sections of the SC are characterized by relatively short statements that document the patient’s need for sick leave. The average number of words on each sickness certificate was 52, with a median of 36. The highest number of words on a single SC was 353, and the lowest was 8 (2 repetitions of the sentence “*admission detoxification four times”.)* Most of the texts had repetitions of the same bulk of texts across the different headings in the SC template, inflating the true value of median and average numbers of words and the amount of information transferred. The certificates had frequent errors, including spelling mistakes, punctuation issues, and inconsistent use of abbreviations. Moreover, we observed that GPs often copied the same text into multiple free-text fields, regardless of the distinct prompts. As a result, the actual transfer of information may be less than what the word count suggests. Possible explanations for the repetition could be time constraints, uncertainty about what information is expected in each field, or perceived redundancy of the topics.

### Tool 1. The subject tool: symptoms, treatment and limitations to work ability

The subject tool examines how SCs frame and prioritize key subjects, such as limitations, symptoms, and ongoing treatment. By analyzing which aspects are emphasized and which are downplayed, this tool can provide insights into how SCs guide the reader’s interpretation. We see a pattern in which the SCs often constructs a narrative that highlights incapacity while overlooking possibilities for recovery, adaptation, or support.

#### Symptoms

As can be expected, psychological symptoms are a dominant subject within SCs, and these are often described in ways that emphasize their impact on daily functioning and work capacity. Less attention is given to strategies for management or recovery.

*“Dark periods in addition to self-destructive behavior – mostly related to food, but also to sleep and circadian rhythm.”* (SC 134)

*“Increasing anxiety, isolation over the past couple of years. Since [date], debilitating and unable to manage work…”* (SC 140)

*“Reduced. Weakened. Concentration, sleep. Panic attacks. Difficult position with employer.”* (SC 98)

These descriptions focus on the breadth and severity of symptoms, constructing a narrative centered on impairment. The fragmented and terse phrasing in SC 98, provides a list of symptoms, but omits any details, reference to treatments, coping mechanisms, or the patient’s potential to adapt or improve, further emphasizing incapacity.

#### Ongoing treatment

In addition to symptoms, SCs frequently highlight ongoing treatment, framing it as necessary and often prolonged, which can be interpreted as positioning the patient’s current state as incompatible with immediate work capacity.

*“Goes to treatment in District Psychiatric Centre [Name]. Recently diagnosed with bipolar disorder after many years of anxiety and depression. Has started medication treatment.”* (SC 105)

*“100% on sick leave since [Date]. Classic depression with lack of initiative, fatigue, and low mood. Assessed at the acute psychiatric outpatient clinic. Started treatment with a psychologist, also undergoing evaluation for ADHD/Dyslexia, but substance use history…”* (SC 92)

These excerpts illustrate the complexity of the patient’s condition and the initiation of treatment as necessary and in process. By prioritizing the topic of ongoing treatment, the GPs implicitly present the topic of work participation as less relevant. The SCs rarely include reflections regarding strategies that could facilitate a gradual return to work or modification to workplace tasks.

#### Limitations to work ability

The patient’s work limitations are also frequently foregrounded in SCs, often presented in definitive terms that underscore the severity of their incapacity.

*“Her depression affects her concentration, her ability to focus on work tasks.”* (SC 132)

*“Because of the situation, the patient is not fit to do work tasks, as her psychiatric symptoms are still deemed to be strong after the incident.”* (SC 120)

These SCs explicitly link the patient’s mental health condition to specific work-related challenges, framing mental health symptoms as a significant barrier to essential job functions like concentration and focus.

By focusing on incapacity without addressing opportunities for adaptation or support, the SCs reinforce a presentation of the patient’s inability to work rather than exploring pathways to potential reintegration to the workplace.

### Tool 2. The vocabulary tool: generalized labels and medical terminology

The vocabulary tool focuses on analyzing the choice of vocabulary and how this contributes to specific patterns in the presentation of the patient’s needs. These choices might play a significant role in the availability of the information and the interpretation related to welfare decisions.

SCs frequently employ medical terminology to describe symptoms, diagnoses, and treatments. Examples include terms such as:

*“chronic anxiety disorder”* (multiple texts), *panic attacks”* (multiple texts), “*PTSD”* (SCs 59, 90, 152), *“hyperemesis gravidarum”* (SC 60), *“OSAS”* (obstructive sleep apnea syndrome, SC 70), and *“conization”* (SC 75)

These terms are often presented as self-explanatory, they are rarely explained or clarified. For example, “OSAS” might signal significant health challenges related to sleep, yet without context or explanation, its relevance to work capacity remains unclear. While appropriate for clinical communication, such language places a burden on readers from non-medical disciplines, who may lack the requisite expertise to fully grasp its implications.

Another key feature of SCs is the reliance on generalized labels, such as diagnoses, to convey complex health conditions:

*“Gradually developed long-term issues with depression and anxiety, occasionally panic attacks. Leads to poor sleep, appetite, and motivation, as well as social withdrawal* (SC 4)

This example provides a relatively comprehensive description, offering insights into the patient’s mental health challenges and their functional implications. However, even here, it remains implicit how these symptoms specifically affect the patient’s work capacity. This highlights both the strengths and limitations of generalized descriptions in SCs – they might convey a comprehensive? picture of the patient’s condition but leave details open to interpretation.

*“Long-term psychological difficulties with trauma, adjustment disorder, anxiety, and depression. Emotional instability found, with impacted sleep, concentration, and appetite.”* (SC 35)

This SC provides a detailed summary of the patient’s mental health challenges, touching on trauma, adjustment disorder, and depression. However, while it highlights broad functional impacts such as emotional instability and disrupted sleep, it does not specify how these impairments affect job tasks. Without such information, the text risks homogenizing both diagnoses and individuals. This lack of nuance undermines the personalized understanding necessary for effective welfare assessments and tailored interventions, potentially perpetuating inequities in support measures.

### Tool 3. The significance building tool: epistemic certainty in diagnosis and prognosis

The Significance Building Tool examines how language in SCs emphasizes or downplays key aspects of the patient’s condition by conveying different levels of epistemic certainty. The analysis shows that SCs often exhibit a high degree of epistemic certainty when discussing diagnoses and limitations, while expressing a low degree of epistemic certainty regarding recovery and possibilities for work.

#### Epistemic certainty in diagnoses and limitations

In many SCs, definitive language conveys a high degree of certainty about the patient’s incapacity:

*“Significant anxiety disorder…”* (SC 123)

*“Struggling with anxiety and depression”* (SC 36)

*“Prolonged mental strain, extremely challenging situation for the family…* (SC 40)

*“Pronounced anxiety and depression…”* (SC 100)

*“Severe anxiety disorder…”* (SC 61)

*“Deep depression…”* (SC 88)

*“This will lead towards 100% disability pension.”* (SC 110)

Adjectives like “severe”, “pronounced”, “significant” and “deep” heighten the perceived impact of the patient’s condition on daily functioning. Statements such as “100% disability pension” project a future of total incapacity, underscoring the chronic and debilitating nature of the illness. These linguistic choices create a narrative of inevitability, advocating for substantial and sustained support.

Also so-called extreme case formulations are employed in descriptions of limitations, i.e. semantically extreme descriptions, invoking the maximal or minimal properties of objects or events [31].

*“Anxiety and depression symptoms hindering any kind of work…”* (SC 129)

The phrase *“any kind of work”* asserts an absolute limitation, conveying strong certainty that the patient’s symptoms prevent engagement in any form of employment. Extreme case formulations are a discursive choice that frequently serve to justify or defend an action as these formulations cut off the basis for further inquiry [32].

*“… Not compatible with driving generally, and of course not with a taxi.”* (SC 70)

The adjective phrase *“not compatible”* denotes absolute incompatibility between the patient’s condition and specific job functions. The addition of the adverb *“of course”* reinforces certainty, suggesting that any other conclusion would be unreasonable.

*“Psychiatric status presens: Avoids eye contact. Appropriate facial expressions. Must stop talking several times to gather their thoughts. Expressionless face. No signs of reality distortion, coherent thought processes. 1-s latency. No sign of psychomotor agitation.* (SC 34)

The GPs observations are presented plainly and objectively, conveying high certainty about the patient’s mental symptoms and signs. The authoritative tone mirrors medical documentation practices, enhancing the perception of factual accuracy.

#### Epistemic uncertainty in recovery and work potential

In contrast, when describing recovery prospects or the patient’s potential to return to work, the language becomes tentative, expressing a degree of uncertainty:

*“…The hope is that it will improve with treatment…”* (SC 70)

The phrase *“the hope is”* clearly indicates uncertainty, framing recovery as a possibility rather than a certainty, reducing expectations of imminent improvement.

*“… hope ability to work will be better once she starts working”* (SC 88)

The word “*hope”* acknowledges doubt about future work ability. While there’s hope for improvement, the lack of certainty tempers expectations.

*“It is difficult to say when she will be able to return to work.”* (SC 88)

This statement explicitly acknowledges uncertainty regarding the patient’s recovery timeline, emphasizing the unpredictability of improvement.

The contrast between epistemic certainty in limitations and uncertainty in recovery contributes to shaping the reader’s interpretation of the patient’s situation. While definitive language about the patient’s current symptoms conveys the severity of the condition, assessing the likelihood or timeline of recovery is inherently more speculative. GPs can observe and record present clinical signs with relative confidence but forecasting an individual’s future course is far less straightforward. This inherent unpredictability naturally leads to more tentative language, since GPs are neither statisticians nor oracles. Consequently, the tension between describing present incapacities with certainty and discussing the future in uncertain terms reflects both the limits of clinical foresight and the practical constraints of sickness certification.

By foregrounding incapacity with certainty and addressing recovery with uncertainty, the SCs might be seen to gain rhetorical function that serve to legitimize the decision to provide sick leave to the patient.

### Tool 4. The figured worlds tool: temporal aspects of recovery

The Figured Worlds Tool examines how language in SCs constructs social realities, in this case particularly by emphasizing the temporal aspects of mental health recovery. In these texts, mental health conditions are often constructed using metaphors and figurative language that depict recovery as a gradual and time-intensive process. This framing shapes the reader’s perception of the patient’s availability for work, creating a narrative where improvement is tied to patience and prolonged support.

*“…needs more time…”* (SC 122)

*“…is in slow recovery, but this will take time…”* (SC 28)

*“Will improve but takes time”* (SC 82)

*“…The patient needs time to build herself up. She is improving…”* (SC 73)

*“Will need full sick leave for quite some time yet”* (SC 96)

These expressions construct a reality where recovery is viewed as inherently lengthy, implicitly signaling delays in the patient’s return to work. Such wording may reflect the clinical reality that mental health conditions often require lengthy recovery periods, yet it can also shape expectations for prolonged work absence.

*“Patient is undergoing treatment for substance-related health issues and needs first to get her cannabis use under control before she can return to ordinary working life.”* (SC 149)

The modal verb “needs” and the condition “before she can return” create a mandatory sequence of events, framing work absence as a prerequisite to recovery. The language performs the dual act of diagnosing incapacity and advocating for extended leave due to ongoing treatment requirements.

The repeated emphasis on time not only constructs recovery as a passive process but may also influence how SCs are interpreted by NAV social workers. Statements like “needs more time” and “will need sick leave for quite some time yet” may lead to expectations of extended incapacity, shaping welfare assessments and resource allocation. This focus on temporal elements risks overshadowing opportunities for workplace accommodations or interventions that might accelerate recovery.

By creating a figured world where mental health recovery depends heavily on the passage of time, these linguistic choices emphasize limitations over strengths. This framing ultimately affects how illness and recovery are perceived, potentially reinforcing narratives of incapacity and influencing institutional decisions.

### Tool 5. The fill-in tool: implied causal relationships

This tool examines the gaps in SCs, focusing on what is explicitly stated versus what the reader must infer to understand the GPs assessments and the implications for work capacity. SCs often rely on brief descriptions, specialized terminology, and implied causal relationships, which might create interpretive challenges, particularly for non-medical readers.

#### Brief descriptions and lack of context

SCs often provide minimal background information, requiring readers to infer critical connections between symptoms, diagnoses, and work capacity. For instance:

*“She goes to counselling due to mental health challenges. Therefore, unable to work due to high burden.”* (SC 2)

In this example, “mental health challenges” is not elaborated upon, leaving readers with no insight into the nature or severity of the condition. Similarly, “high burden” is vague, potentially encompassing physical, emotional, or cognitive strain – or a combination of all three. Without specific details, the readers are left to fill in these gaps, increasing the likelihood of varied interpretations.

Similarly:

*“Cannot manage anything, does not know where to start – but also during today’s consultation spontaneously mentions anxiety issues.”* (SC 6)

This SC provides a broad depiction of incapacity, using extreme case formulations like “cannot manage anything”, but lacks details about how these struggles affect work tasks. The mention of anxiety is similarly underexplored, offering little context or depth about its impact.

Many SCs imply causal links between diagnoses and work incapacity without explicitly articulating them. For example:

*“…Suffered a brain infarction following surgery, caused by an embolus from her artificial aortic valve. Is on Marevan…”* (SC 48)

The inclusion of “Marevan” (a blood thinner) assumes that the reader understands its medical implications, such as whether it has risks or may restrict certain work activities. However, without further context, the burden of interpretation shifts to the reader.

*“She has always been emotionally unstable; this worsened significantly after the birth; developed anxiety and panic attacks…”* (SC 60)

This sentence implies a causal link between childbirth, emotional instability, and anxiety but fails to specify the functional consequences. Many of the SCs lack this information that may add complexity to interpretation, particularly for non-medical readers unfamiliar with psychiatric reasoning.

#### Elliptical style

Elliptical constructions are common in SCs, i.e. sentences in which one or more words are omitted, and the meaning must be implied by the reader.

*“Perceived worsening of ulcerative colitis. After psychological destabilization following violence and psychological abuse.”* (SC 1)

The elliptical phrasing suggests a connection between psychological trauma and physical health deterioration but does not include crucial details about causality, symptoms, and timelines. This lack of explicitness leaves readers to infer connections.

Additionally, syntactic simplicity can lend authority but risks oversimplifying complex conditions:

*“Felt nervous, anxious, or very stressed. Unable to stop worrying or control their worries. Worried too much about various things. Had difficulty relaxing. Been so restless that it was hard to sit still. Easily angered or irritated, felt afraid as if something terrible might happen.”* (SC 25)

While this condensed style conveys factual certainty, it again lacks nuance to how it affects work capacity. This reliance on brevity and implicit connections reflects a “medical journal style”, which may prioritize clinical efficiency over interdisciplinary clarity.

### Tool 6. The doing and not just saying tool: justifying work absence

This tool explores how language in SCs functions not merely to describe the patient’s condition but to advocate for specific outcomes, such as continued treatment and work absence.

The language often employs definitive statements and strong modal verbs, framing work absence as undisputable.

*“Not possible to combine with activity.”* (SC 123)

A number of SCs simply state *“Cannot attend work.”* (Multiple SCs: 34, 38, 48, 49, 69, 75, 80, 81, 83, 87, 99, 107, 114, 118, 120, 125, 129, 137, 149 and 152).

Statements like these assert incapacity with finality. Phrases such as “not possible” and “cannot attend work” eliminate the consideration of partial work ability. These patterns suggest that the SCs often function as documentation of the GPs assessment and not as an invitation to NAV to investigate further or explore opportunities for the patient. The rhetorical aspects of the SCs become apparent with this tool, as the SCs descriptions effectively preclude other options than work absence.

#### Using patient history to legitimize incapacity

In addition to the factual and assertive style, references to the patient’s medical history or their specific circumstances also serve to strengthen the justification for continued absence from work.

*“Long-standing psychological overload, extremely demanding family situation with a father suffering from severe brain damage, reactive depression.”* (SC 40)

By emphasizing the temporal dimension (“long-standing”) and the boosted description of the family situation (“extremely demanding”), the SC highlights contextual details that strengthens and legitimizes work absence, framing the patient’s incapacity as both severe and unavoidable.

*“She has had many psychological traumas, got a severe depression last winter. Also, very bad shoulders.”* (SC 152)

This statement layers multiple health issues – psychological trauma, severe depression and physical ailments – creating a comprehensive narrative of incapacity. The accumulation of challenges strengthens the rhetorical case for extended absence from work.

These rhetorical strategies align with institutional goals of advocating for patient needs but may risk presenting a one-sided view that does not address NAV concerns that relate to work ability and options for continued work participation. As Aarseth et al. [33] note, such a selective framing supports the patient’s claim for sick leave but may inadvertently skew assessments toward incapacity, potentially shaping welfare decisions and resource allocation.

### Tool 7. The collaboration tool: absence of reference to cross-institutional collaboration

The collaboration tool explores how SCs make relevant collaboration between institutions such as NAV, healthcare providers, and other stakeholders. Despite NAV specifically requesting input on this topic in Sects. 7.2 (*NAV measures. Describe)* and 7.3 *(Other suggestions for NAV. Describe)*, language that orient to this collaboration is strikingly underrepresented in most responses. These sections, designed to encourage actionable recommendations, contain minimal information.

Responses often contain vague or generic statements, offering little insight into how NAV or other stakeholders might assist the patient towards return to work. The sparse utilization of these sections may reflect a lack of practical guidance, but also lack of information about the patient’s workplace, or possibilities and structures within NAV.

While exceptions exist, they remain rare and highlight missed opportunities for a more structured, collaborative approach. For instance:

*“He must himself contact NAV to plan further steps…”* (SC 24)

The responsibility is placed on the patient to initiate contact and explore possibilities.

In contrast, another GP writes:

*“I believe NAV should contact the patient regarding help with job placement or training…”* (SC 127)

This SC explicitly suggests that NAV take an active role in supporting the patient through practical measures, such as job training or placement.

*“It is absolutely necessary to have a collaborative meeting between DPS [District Psychiatric Centre], NAV, [Municipality], and the doctor to plan further steps.”* (SC 115)

This rare example highlights the potential for a structured, multi-institutional collaboration to address complex patient needs.

Despite these notable examples, the overall lack of references or invitations to collaboration suggests that GPs rarely engage with NAV’s explicit invitation to give input on actionable recommendations in the form. This absence raises questions about whether GPs see their role as extending to broader institutional collaboration or if systemic factors, such as time constraints or lack of clarity about expectations, contribute to this gap.

## Discussion

This study underscores how the language and structure of SCs often foreground incapacity over recovery potential, shaping perceptions of patient’s work capacity in a particular way. These narratives do not merely steer administrative decisions; they may also influence patient’s own outlook on recovery and work participation, reinforcing or challenging their sense of agency [17]. The analysis reveals that specific discursive choices – such as definitive statements, extreme-case formulations, and elliptical language – can guide readers to emphasize limitations. However, SCs are crafted within a complex, time-pressured environment where GPs must navigate between medical accuracy, patient expectations and administrative demands. The systemic constraints, alongside resource limitations, likely shapes the depth of information conveyed in SCs, often resulting in content that may lack the detail NAV’s decision-makers require.

### Time constraints and information gaps in sickness certification

A central challenge for GPs is the need to complete SCs concisely within limited consultation times. GPs are expected to diagnose, assess functional capacity, and document all relevant information efficiently, often within tight time constraints (on average 20 min per consultation in Norway) that restricts the opportunity to provide nuanced details about a patient’s work capacity or recovery prospects [34]. These time pressures naturally limit the depth of detail that can be provided, probably leading to SCs that rely on broad terminology and shorthand. This reliance on brevity obscures critical context, especially when SCs omit specifics about the patient’s functional limitations or potential for adaptation.

For non-specialist readers, such as NAVs social workers, this lack of specificity presents interpretative challenges – broad terms and elliptical language require non-specialists to infer link between symptoms and functional capacity, which may not always be explicit [35]. This variability in language reflects an implicit assumption that SC readers possess a shared medical knowledge, which may not align with interdisciplinary realities. Broad, generalized labels often fail to address how symptoms translate into workplace limitations or adaptation needs.

Beyond logistical pressures, GPs also face an emotional burden in situations where they feel compelled to withhold an SC. Nilsen and Malterud [24] emphasize that GPs experience significant stress in these situations, as they balance responsibilities with patient expectations within a constrained healthcare system. Together, these practical and emotional pressures impact SC content, often limiting the information NAV receives and complicating decision-making.

A recurring feature across the SCs is the absence of discussions about the patient’s potential abilities, available resources, or opportunities for adaptation. The focus remains fixed on limitations, symptoms, and treatment, often excluding mentions of workplace accommodations, rehabilitations programs, or other supportive measures. One explanation is that GPs often lack detailed knowledge of patients’ specific job demands or see workplace accommodations as beyond their scope, leading them to focus primarily on medical diagnoses. As a result, NAV social workers – without clear information on remaining capacities – may default to more conservative decisions that effectively prolong sick leave.

The frequent omission of responses in section 7.1 reveals a challenge in the SC process. This section requires GPs to recommend workplace accommodations, yet they often lack the necessary knowledge of the patient’s job duties, work environment, or the feasibility of proposed adjustments. Even when responses were provided, they varied significantly, complicating efforts to implement effective support measures. This highlights the need for clearer distinction as to who should answer what, and a probable need for improved collaboration between GPs, patients, NAV and employers.

### Navigating conflicting pressures: GPs as frontline decision-makers

Lipsky’s theory of street-level bureaucracy [20] provides a useful framework for understanding the complex role of the GPs in creating SCs. Street level bureaucrats, such as GPs [21], operate in public service roles with direct citizen (patient) interaction, substantial autonomy, considerable discretion, limited resources, high demand, and conflicting expectations. In documenting SCs, GPs must exercise professional judgement to determine which details to emphasize and how to represent the patient’s condition, balancing clinical relevance with what they perceive NAV may require for decision-making purposes ([22], pp. 940–949). This discretion allows GPs to tailor SCs to individual patient needs and circumstances, but it also introduces a natural variability in documentation practices.

Zacka [23] expands on Lipsky’s concepts by examining the ethical and emotional dimensions of discretion in frontline bureaucratic roles. Zacka highlights that street-level bureaucrats, including GPs, face moral dilemmas when their professional responsibilities intersect with the personal impact of their decisions on clients. This discretionary decision-making is often constrained by structural pressures and resource limitations, which may limit how fully they can document all relevant aspects of a patient’s condition.

Zacka’s insights may be particularly relevant to SCs, where GPs may feel compelled to represent the patient’s incapacity in more definitive terms, partly due to the perceived demands of NAV or the expectations of the patient. Ethical tension arises when GPs must choose between documenting a simplified, perhaps overly generalized assessment and providing a more nuanced description that may open for a denial of benefits from NAV.

GPs, as street-level bureaucrats (Lipsky), continually manage the dual responsibility of maintaining clinical accuracy and upholding institutional requirements, often under less-than-ideal conditions. This will affect the consistency of SCs, as different GPs may emphasize different aspects of a patient’s condition depending on their own interpretation of NAV’s needs, their relationship with the patient, or the time available. This underscores the structural challenges in producing standardized SCs that are interpretable across interdisciplinary contexts.

### Implications for NAV and interdisciplinary communication

Our findings indicate that the current SC format and content may not consistently meet NAV’s needs for relevant and actionable information, especially when functional abilities are vague or ambiguous [11]. NAV relies on SCs for making informed decisions about eligibility and support measures, but interpretative gaps can lead to inconsistency in assessments and threaten equal right to services.

The findings from the collaboration tool reveal a significant gap in how SCs function to facilitate interdisciplinary efforts. Despite sections explicitly requesting actionable recommendations, such as NAV measures or workplace accommodations, responses were often vague, inconsistent, or absent altogether. This does not necessarily reflect a lack of effort by GPs but rather the inherent challenge of answering questions that lie outside their scope of expertise. GPs, as medical professionals, may lack detailed knowledge of workplace environments or feasible accommodations. SCs seem to place a disproportionate burden on GPs to translate medical conditions into work-related recommendations, often without the support or expertise required for such interdisciplinary evaluations. Addressing this limitation may require a structural overhaul of the SC process, ensuring workplace-specific assessments are conducted collaboratively with input from employers, NAV specialists and the patient, rather than relying mostly on GPs.

To improve the relevance and utility of SCs, a broader strategy than simply modifying SC formats may be required. While reducing reliance on medical jargon and providing more structured prompts could help GPs highlight functional impairments, engaging employers and patients in the documentation process would ensure NAV receives relevant workplace details. Guo [36] highlights the importance of interdisciplinary texts that bridge professional silos. A more interdisciplinary approach, incorporating employers alongside healthcare providers, could foster a well-rounded perspective on a patient’s work capacity, thereby both aiding NAV and relieving GPs. In this instance, the SC’s role would be less of a conclusion sent to NAV, but more a collaborative tool for patient support [37, 38].

### Limitations and future research

While this study provides insights into the linguistic and structural challenges of SCs, it is limited in scope due to sample size and focus on specific discursive strategies. Future research could explore what kinds of SC content has value and relevance for NAV social. Additionally, examining how variations in GP training or differing interpretations of NAV guidelines influence SC content would add depth to the understanding of discretionary practices. Future research could benefit from data sets that includes a variety of medical conditions and SC formats to better understand how different content types and discursive choices influence NAV’s assessments.

Gee acknowledges that his discourse analysis framework is primarily qualitative, potentially limiting its applicability in research contexts requiring quantitative validation. Additionally, he recognizes that discourse analysis may not fully capture broader institutional and social contexts, as it focuses on immediate linguistic interactions ([39], pp. 119–169). Despite these limitations, Gee’s tools remain valuable for examining nuanced meanings within SCs, offering insights into the language that shapes welfare decision-making.

Furthermore, while this study highlights the discretionary role of GPs, future research could delve into examining the influence of interdisciplinary collaboration, such as incorporating direct input from employers. Further research could investigate how this more holistic approach could be standardized and effectively integrated into the SC process, and if this may support development of a more collaborative, patient-centered framework.

## Conclusion

This study has shown that SCs often emphasize incapacity, which may skew interpretations towards limitations over recovery potential. The discourse analytic approach reveals how SCs communicate assumptions about patient functionality and capacity, and underscores the value of studying actual SCs that serve as basis for welfare decisions.

Through the lens of Lipsky’s theory of street-level bureaucracy and Zacka’s exploration of ethical discretion, we see that GPs exercise professional judgment within an environment of limited resources and conflicting demands, which may naturally introduce variability in SC content.

Addressing these challenges requires structural changes to SCs that promote relevance, clarity and accessibility. Suggestions include revising SC formats to encourage clearer articulation of functional impairments and specific accommodations and fostering interdisciplinary approaches involving employers, NAV, and patients. Rather than viewing SCs as standalone medical assessments, a collaborative framework would enhance their role as tools for patient recovery planning.

In order to access relevant information of functional ability for specific patients in specific workplaces, employer accountability could be strengthened by encouraging employers to take an active role in assessing and facilitating accommodations. Incorporating input from employers and NAV into SC evaluations could provide contextual insights that GPs lack, thereby bridging the gap between medical assessments and workplace realities.

Ultimately, it’s not just about the certificates that are written, but the stories they tell – shifting from narratives of incapacity to ones of potential can transform SCs from isolated medical records into catalysts for recovery and reintegration.

## Supplementary Information

Supplementary Material 1. Translated version for the Norwegian sickness certificate form (NAV 08–07.4), referenced in the study.

## Data Availability

The datasets analyzed during the current study are not publicly available due to privacy and confidentiality restrictions.
