# Supplementary material for: Legitimizing incapacity: discursive choices in Norwegian sickness certificates
Source: BMC Health Serv Res. 2025 May 20;25:725. doi: 10.1186/s12913-025-12902-7 (PMC12090561; doi:10.1186/s12913-025-12902-7)
Supplement: Supplementary file 1 — Supplementary Material 1. Translated version for the Norwegian sickness certificate form (NAV 08–07.4), referenced in the study. [file 12913_2025_12902_MOESM1_ESM.docx]

Supplementary material

Sickness certificate week 39

| **Norwegian (Original)** | **English (Translation)** |
| --- | --- |
| **VURDERING AV ARBEIDSMULIGHET / SYKMELDING VED 39 UKER** *Fødselsnummer* | **ASSESSMENT OF WORK CAPABILITY / SICK LEAVE AT 39 WEEKS** *Personal ID number* |
| 0. Når startet det legemeldte fraværet? Annen lovfestet fraværsgrunn § 8-4, 3. ledd – oppgis hvis relevant | 0. When did the medically certified absence begin? Other statutory absence reason § 8-4, third paragraph – provide if relevant **(Date; specify reason if applicable)** |
| **1 Pasientopplysninger** | **1 Patient information** |
| 1.1.1 Etternavn | 1.1.1 Last name *(text field)* |
| 1.1.2 Fornavn | 1.1.2 First name *(text field)* |
| 1.2 Fødselsnummer | 1.2 Personal ID number *(11-digit national ID)* |
| 1.3 Telefon | 1.3 Telephone *(phone number)* |
| 1.4 Navn på pasientens fastlege | 1.4 Name of patient’s regular doctor (GP) *(text)* |
| 1.5 NAV-kontor | 1.5 NAV office *(text)* |
| **2 Arbeidsgiver** | **2 Employer** |
| 2.1 Pasienten har:   ☐ arbeidsgiver   ☐ flere arbeidsgivere   ☐ ingen arbeidsgiver | 2.1 The patient has:   ☐ an employer   ☐ multiple employers   ☐ no employer **(check one)** |
| 2.2 Arbeidsgiver for denne sykmeldingen | 2.2 Employer for this sick leave *(text field: name of employer)* |
| 2.3 Yrke/stilling for dette arbeidsforholdet | 2.3 Occupation/position for this employment *(text field)* |
| 2.4 Stillingsprosent | 2.4 Position percentage *(% of full-time)* |
| **3 Diagnose** | **3 Diagnosis** |
| 3.1 **Hoveddiagnose** – 3.1.1 Kodesystem | 3.1 **Main diagnosis** – 3.1.1 Code system *(e.g. ICD-10/ICPC-2)* |
| 3.1.2 Kode | 3.1.2 Code *(diagnosis code)* |
| 3.1.3 Diagnose | 3.1.3 Diagnosis *(text description)* |
| **Bidiagnoser** – 3.2.1 Kodesystem | **Secondary diagnoses** – 3.2.1 Code system |
| 3.2.2 Kode | 3.2.2 Code |
| 3.2.3 Diagnose | 3.2.3 Diagnosis *(text, optional)* |
| 3.3.1 Lovfestet fraværsgrunn | 3.3.1 Statutory absence reason *(if applicable)* |
| 3.3.2 Beskriv fraværsgrunn (valgfritt) | 3.3.2 Describe absence reason (optional) *(text)* |
| 3.4 ☐ Sykdommen er svangerskapsrelatert | 3.4 ☐ The illness is pregnancy-related **(check if yes)** |
| 3.5 ☐ Sykmeldingen kan skyldes en yrkesskade/yrkessykdom | 3.5 ☐ The sick leave may be due to an occupational injury/illness **(check if yes)** |
| 3.6 Eventuell skadedato: | 3.6 Injury date, if applicable: *(Date)* |
| 3.7 ☐ Det er påtrengende nødvendig å skjerme pasienten for medisinske opplysninger, jf. pasient- og brukerrettighetsloven §§ 3-2 og 5-1 | 3.7 ☐ It is urgently necessary to shield the patient from medical information, cf. Patient Rights Act §§ 3-2 and 5-1 **(check if applicable)** |
| **4 Mulighet for arbeid** | **4 Ability to work** |
| 4.1 ☐ Pasienten kan benytte avventende sykmelding | 4.1 ☐ The patient can utilize a pending sick leave **(check if yes)** |
| 4.1.1 f.o.m. | 4.1.1 from (start date) |
| 4.1.2 t.o.m. | 4.1.2 to (end date) |
| 4.1.3 Innspill til arbeidsgiver om tilrettelegging | 4.1.3 Input to employer about work accommodations *(free-text field for suggestions)* |
| 4.2 ☐ Pasienten kan være delvis i arbeid (gradert sykmelding) | 4.2 ☐ The patient can work partially (graded sick leave) **(check if yes)** |
| 4.2.1 f.o.m. | 4.2.1 from (date) |
| 4.2.2 t.o.m. | 4.2.2 to (date) |
| 4.2.3 Oppgi grad for sykmelding | 4.2.3 Specify degree of sick leave *(percent exempted)* |
| 4.2.4 ☐ Pasienten kan være i delvis arbeid ved bruk av reisetilskudd | 4.2.4 ☐ The patient can work partially by using a travel subsidy **(check if applicable)** |
| 4.3 ☐ Pasienten kan ikke være i arbeid (100 % sykmelding) | 4.3 ☐ The patient cannot work (100% sick leave) **(check if yes)** |
| 4.3.1 f.o.m. | 4.3.1 from (date) |
| 4.3.2 t.o.m. | 4.3.2 to (date) |
| 4.3.3 Medisinske årsaker hindrer arbeidsrelatert aktivitet. Hvis ja… | 4.3.3 Medical reasons prevent work-related activity. If yes… **(check if true)** |
| **Annet:** ☐ Helsetilstanden hindrer pasienten i å være i aktivitet ☐ Aktivitet vil forverre helsetilstanden ☐ Aktivitet vil hindre/forsinke bedring av helsetilstanden ☐ Forhold på arbeidsplassen vanskeliggjør arbeidsrelatert aktivitet. Hvis ja:    ☐ Manglende tilrettelegging    ☐ Annet    Beskriv nærmere *(Kan leses av arbeidsgiver)* | **Other reasons:** ☐ The health condition prevents the patient from being active ☐ Activity will worsen the health condition ☐ Activity will hinder/slow improvement of the health condition ☐ Conditions in the workplace make work-related activity difficult. If yes:    ☐ Lack of accommodation    ☐ Other    Describe in more detail *(Can be read by employer)* |
| 4.4 ☐ Pasienten kan ikke være i arbeid på behandlingsdager | 4.4 ☐ The patient cannot work on treatment days **(check if yes)** |
| 4.4.1 f.o.m. | 4.4.1 from (date) |
| 4.4.2 t.o.m. | 4.4.2 to (date) |
| 4.4.3 Oppgi antall dager i perioden | 4.4.3 Specify number of days in this period *(number of days)* |
| 4.5 ☐ Pasienten kan være i fullt arbeid ved bruk av reisetilskudd | 4.5 ☐ The patient can work fully by using a travel subsidy **(check if yes)** |
| 4.5.1 f.o.m. | 4.5.1 from (date) |
| 4.5.2 t.o.m. | 4.5.2 to (date) |
| **5 Friskmelding/Prognose** | **5 Recovery/Prognosis** |
| 5.1 ☐ Pasienten er 100 prosent arbeidsfør etter denne perioden | 5.1 ☐ The patient will be 100 percent fit for work after this period **(check if yes)** |
| 5.1.1 Beskriv eventuelle hensyn som må tas på arbeidsplassen | 5.1.1 Describe any considerations that must be taken at the workplace *(free-text field)* |
| 5.2 **Pasient med arbeidsgiver:** Utdypende opplysninger ved 39 uker | 5.2 **Patient with employer:** Additional information at 39 weeks |
| – Jeg antar at pasienten på sikt kan komme tilbake til samme arbeidsgiver | – I assume that the patient can eventually return to the same employer. **(If yes, complete 5.2.1)** |
| 5.2.1 Anslå når du tror dette kan skje | 5.2.1 Estimate when you think this can happen *(date or time frame)* |
| 5.2.2 ☐ Jeg antar at pasienten på sikt kan komme i arbeid hos annen arbeidsgiver | 5.2.2 ☐ I assume that the patient can eventually work for a different employer **(check if applicable)** |
| 5.2.3 Hvis usikker: Når antar du å kunne gi tilbakemelding på dette? | 5.2.3 If uncertain: When do you expect to be able to give feedback on this? *(date or time frame)* |
| 5.3 **Pasient uten arbeidsgiver:** Utdypende opplysninger ved 39 uker | 5.3 **Patient without employer:** Additional information at 39 weeks |
| – Jeg antar at pasienten på sikt kan komme tilbake i arbeid | – I assume that the patient can eventually return to work (in general). **(If yes, complete 5.3.1)** |
| 5.3.1 Anslå når du tror dette kan skje | 5.3.1 Estimate when you think this can happen *(date or time frame)* |
| 5.3.2 Hvis usikker: Når antar du å kunne gi tilbakemelding på dette? | 5.3.2 If uncertain: When do you expect to be able to give feedback on this? *(date or time frame)* |
| **6 Utdypende opplysninger** | **6 Detailed information** |
| 6.5 **Helseopplysninger mot slutten av sykepengeperioden** | 6.5 **Health information toward the end of the sick-leave period** |
| 6.5.1 Beskriv kort sykehistorie, symptomer og funn i dagens situasjon | 6.5.1 Describe briefly the medical history, symptoms, and findings in the current situation *(text field)* |
| 6.5.2 Hvordan påvirker dette funksjons-/arbeidsevnen? | 6.5.2 How does this affect the patient’s functional ability/work capacity? *(text field)* |
| 6.5.3 Beskriv pågående og planlagt henvisning, utredning og/eller medisinsk behandling | 6.5.3 Describe ongoing and planned referral, investigation and/or medical treatment *(text field)* |
| 6.5.4 Kan arbeidsevnen bedres gjennom medisinsk behandling og/eller arbeidsrelatert aktivitet? I så fall hvordan? Angi tidsperspektiv. | 6.5.4 Can work ability be improved through medical treatment and/or work-related activity? If so, how? Indicate time frame. *(text field)* |
| 6.6 **Helseopplysninger dersom pasienten søker om arbeidsavklaringspenger** *(Husk at pasienten selv må søke om AAP)* | 6.6 **Health information if the patient applies for Work Assessment Allowance** *(Remember that the patient must apply for AAP themselves)* |
| 6.6.1 Hva antar du at pasienten kan utføre av eget arbeid/arbeidsoppgaver i dag eller i nær framtid? | 6.6.1 What do you believe the patient can currently do in terms of their own work/tasks, or in the near future? *(text field)* |
| 6.6.2 Hvis pasienten ikke kan gå tilbake til eget arbeid, hva antar du at pasienten kan utføre av annet arbeid/arbeidsoppgaver? | 6.6.2 If the patient cannot return to their own work, what do you believe the patient can do in terms of other work/tasks? *(text field)* |
| 6.6.3 Hvilken betydning har denne sykdommen for den nedsatte arbeidsevnen? | 6.6.3 What significance does this illness have for the reduced work ability? *(text field)* |
| **7 Hva skal til for å bedre arbeidsevnen?** | **7 What is needed to improve work ability?** |
| 7.1 Tilrettelegging/hensyn som bør tas på arbeidsplassen. **Beskriv** *(Kan leses av arbeidsgiver)* | 7.1 Accommodations/considerations that should be made at the workplace. **Describe** *(Can be read by employer)* *(text field)* |
| 7.2 Tiltak i regi av NAV. **Beskriv.** *(Hvis det er behov for bistand fra NAV nå, bruk felt 8.)* | 7.2 Measures under the direction of NAV. **Describe.** *(If assistance from NAV is needed now, use field 8.)* *(text field)* |
| 7.3 Eventuelle andre innspill til NAV. **Beskriv.** | 7.3 Any other suggestions to NAV. **Describe.** *(text field)* |
| **8 Melding til NAV** | **8 Message to NAV** |
| 8.1 Ønskes bistand fra NAV nå? | 8.1 Is assistance from NAV wanted now? *(Yes/No)* |
| 8.2 Beskriv nærmere | 8.2 Describe further *(if yes, free-text details)* |
| **9 Melding til arbeidsgiver** | **9 Message to employer** |
| 9.1 (Fritekst til arbeidsgiver) | 9.1 (Free-text field for employer message) |
| **11 Tilbakedatering** | **11 Backdating** |
| Hvis denne sykmeldingen er tilbakedatert, oppgi dato for dokumenterbar kontakt med pasienten. *(Kan leses av arbeidsgiver)* | If this sick leave is backdated, provide the date of documentable contact with the patient. *(Can be read by employer)* |
| 11.1 Dato for kontakt | 11.1 Date of contact *(Date field)* |
| Pasienten har ikke kunnet ivareta egne interesser. **Begrunn**: | The patient has not been able to safeguard their own interests. **Explain**: *(justify reason for late certification)* |
| 11.2 Begrunnelse | 11.2 Justification *(text field)* |
| **12 Bekreftelse** | **12 Confirmation** |
| 12.1 ☐ Pasienten er kjent eller har vist legitimasjon | 12.1 ☐ The patient is known to the certifier or has shown identification **(check to confirm identity)** |
| **Dato:** ________    **Underskrift:** ________ | **Date:** ________    **Signature:** ________ |
| 12.2 Sykmelders navn | 12.2 Certifier’s name *(text)* |
| 12.4 HPR-nummer | 12.4 HPR number *(physician’s ID number)* |
| 12.5 Telefon | 12.5 Telephone *(contact phone number)* |
| 12.6 Adresse | 12.6 Address *(work address of certifier)* |
